# Inequities in cataract surgical coverage in South Asia

**Published:** 2017-02-10

**Authors:** Rohit Khanna, GVS Murthy

**Affiliations:** 1 Director, Gullapeli Pratibha Rao Internations Centre for Advancement of Rural Eye Care (GPRI CARE), Hyderabad; 2 Vice-President, South, Public Health Foundation of India & Director, Indian Institute of Public Health.

**Figure:**
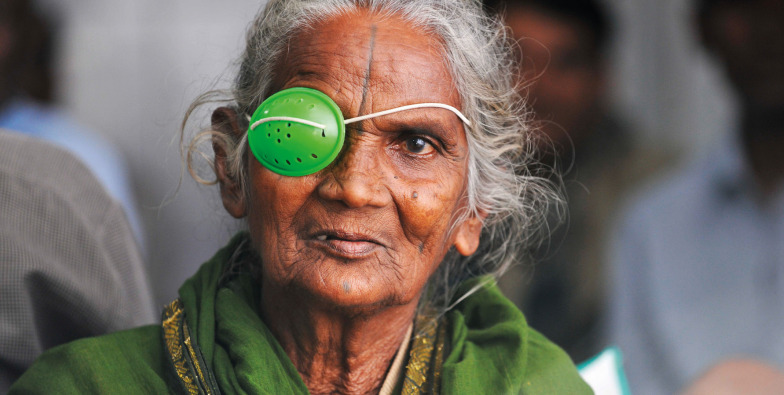
Countries with lower GDP and per capita health expenditure tend to have a higher incidence of inequity in eye care

## Introduction

Recent estimates from the World Health Organization (WHO) show that globally there are 285 visually impaired people of which 39 million are blind.^1^ Cataract is the major cause of blindness and second leading cause for visual impairment (VI).^1^ One of the important parameters to measure the impact of cataract services is the Cataract Surgical Coverage (CSC). CSC is also one of the indicators to monitor the progress of the Universal Eye Health: Global Action Plan 2014-19.^2^ CSC is defined as the proportion of people or eyes with cataract eligible for cataract surgery who have received cataract surgery in at a given point in time.’ It is one of the parameters or measures obtained from the Rapid Assessment of Avoidable Blindness (RAAB) or Rapid Assessment of Cataract Surgical Services (RACSS) studies. It can also be obtained from other population based studies (Table 1 and 2). There is a gross variation in CSC across different countries as well as regions within the same country. Apart from this, gender inequality in CSC has been reported from different low and middle income countries.^3^,^4^ In this article we review the CSC data from countries in South-Asia (SA) and review inequities between and within countries, especially related to gender. We also review the association between country wealth and government health expenditure on CSC i.e. with Gross Domestic Product (GDP) of a country as well as per capita health expenditure. In simple terms, GDP is the total monetary value of all goods and services produced within a nation's geographic borders over a specified period of time. It is a measure of a country's total economic activity. Health expenditure is the sum of public and private health expenditure as a ratio of total population.

## Methods

South Asia encompasses Bangladesh, Bhutan, India, Maldives, Nepal Pakistan and Sri Lanka. CSC data (stratified by gender) was obtained from published literature, the RAAB repository, as well as by personal communications with the Principal Investigators (PI) of some studies. CSC data was available for all countries except Maldives. Of the remaining countries, gender specific data was available for all. Data from Bangladesh, Bhutan, Nepal and Pakistan represented the entire country. Data from Bangladesh, Bhutan and Pakistan were from published sources, while data from Nepal was obtained from the RAAB repository. From other countries, regional data were available. Hence, extrapolation of these regional specific data to the entire country may not be appropriate. The CSC data (person and eyes) from these countries (stratified by gender) is shown in Tables 1 and 2.

## Results

There is a wide variation in terms of people accessing cataract services. For visual acuity level of < 3/60, the range is from 30.5% (Sindhudurg, India) to 92% (Surat, India). At a CSC cut-off level of <6/60 and <6/18 the CSC is naturally lower than at <3/60. For visual acuity < 6/60, the range is 46.8% (Bangladesh) to 85.9% (Srisailam, India) and for visual acuity level <6/18, it was 32.4% (Bangladesh) to 68% (Integrated Tribal Development Agency area of West and East Godavari, India) (Table 1).

Similar trend was seen for CSC for eyes (Table 2). CSC for eyes with the same cut-off of visual acuity (<3/60; <6/60 and <6/18) was lower than for persons suggesting that most of these participants had unilateral cataract surgery.

All the countries had lower CSC for females as compared to males (Table 1 and 2). In countries like Bangladesh, Bhutan and Sri Lanka the difference was high. A similar difference is seen for other levels of visual acuity (<6/60 and <6/18). This suggests a significant inequity in terms of females accessing services for cataract, especially in Bangladesh, Bhutan and Sri Lanka. These countries also reporta lower GDP and per capita health expenditure than the other countries in the region suggesting that in poorer countries, women are less likely to access eye care services compared to economically richer countries. Gender difference could be due to gender-defined social roles, which could be confounded by factors like literacy, socioeconomic status as well as urban-rural differences. It is likely that women in countries with lower CSC are less educated, have other domestic responsibilities and are not the main earning member of the house, thus having less access to eye care as well as other health care services. However, limited data was available in relation to literacy, socio-economic status and urban-rural differences. Data from Bhutan showed that those residing in rural areas had a lower CSC as compared to their urban counterparts^5^. Similarly data from Nepal (Gandaki Zone) showed that CSC was lower in illiterates^6^. A study conducted in Sivaganga also showed that CSC was lower in older people, those with no education as well as those residing in rural areas^7^. Pakistan National Blindness and VI survey also showed lower CSC for illiterates, those residing in rural areas as well as older people, suggesting gross inequity^8^.

## Conclusions and Recommendations

- There is gross inequity in terms of CSC in countries of South Asia i.e. females have less access than males
- Inequity is also compounded due to other social determinants like socio-economic status, literacy, urban-rural difference etc. However, there is limited evidence for it.
- Countries with lower GDP and per capita health expenditure, are likely to have more inequity
- We recommend that there is a need for data to be collected from countries where there is none. In countries where there is only region-specific data, data is needed to be representative of the whole country. Also data including key social determinants need to be collected.
- All countries should work towards achieving the goal of Universal Eye Health with at least 80% CSC for <3/60 visual acuity category as well as ensuring that women, and those from the lower socio-economic strata and rural areas have improved access to services.

## Limitations

One of the limitations of the data is that it is not representative of all the countries. We did not do any analysis to see if the difference between gender was significant or not. There was limited data available in terms of other social determinants (socio-economic status, literacy, urban-rural difference etc).

**Table 1:** Cataract Surgical Coverage (by person), stratified by gender for countries in South Asia

Country	Location	Year	Person	Person	Person	Person	Person	Person	Person	Person	Person	GDP at time of survey*	Per capita health expenditure**
			**Less than 3/60**			**Less than 6/60**			**Less than 6/18**				
			**Male**	**Female**	**Total**	**Males**	**Females**	**Total**	**Males**	**Females**	**Total**		
India^	15 districts in 16 states	2007	NA	NA	82.3	NA	NA	66	NA	NA	NA	$1.23 trillion	$43
India	Nandurbar^9^	2009	NA	NA	NA	NA	NA	NA	NA	NA	NA	$1.36 trillion	$48
India	Kolar^10^	2011	84.6	79.7	81.7	75.7	69.8	72.2	65.6	63.1	64.1	$1.83 trillion	$66
India	Sindhudrug^11^	2010	32	28.4	30.5	NA	NA	NA	NA	NA	NA	$1.17 trillion	$59
India	Sivaganga^7^	1999	NA	NA	NA	80.9	75.2	77.5	NA	NA	NA	$466.86 billion	$18
India^	ITDA-Khammam & Warngal	2009	88.3	87.8	88	79.6	78.8	79.1	62.4	67.2	65.1	$1.36 trillion	$48
India^	ITDA-East Godavari & West Godavari	2009	86.2	83.8	84.6	76.5	78.6	77.8	65.1	69.8	68	$1.36 trillion	$48
India^	ITDA-Srisail-am	2009	95.7	88.1	91.5	90.1	82.6	85.9	68.9	63.6	65.9	$1.36 trillion	$48
India@	Tribal region In Su rat Gujarat	2011	95.7	89.6	92	88.4	79.2	82.7	60.1	51.6	54.9	$1.83 trillion	$66
Bangladesh	Satkhira^12^	2005	63.6	59	60.9	57.9	55.1	56.3	34.5	36.4	35.6	$69.44 billion	$12
Bangladesh	8 districts^13^	2010	76.6	64.3	69.3	51.1	43.9	46.8	35.1	30.5	32.4	$115.27 billion	$23
Bhutan [Urban]	Whole country^5^	2005	81.8	85	83.3	82.6	72	77	60	50	54.7	$818.86 billion	$66
Bhutan [Rural]	Whole country	2005	75	60	67.4	60.7	43.1	51.2	41.4	27.3	34	$818.86 billion	$66
Bhutan [Both]	Whole country	2005	77.8	67.7	72.7	67.1	51.1	58.6	46.3	33.3	39.4	$818.86 billion	$66
Srilanka [40 yrs above and bleow]#	Kandy^14^	2006	90.6	76.7	82.7	80	74.2	76.8	47.3	41.8	41.9	$28.27 billion	$58
Nepal@	Whole country	2008-2010	88	83	85	72	69	70	56	54	55	$12.54 billion	$29
Pakistan#	Whole country^8^	2003-2005	79.6	74.9	77.1	70.1	68.4	69.3	44.6	42.8	43.7	$83.24 billion	$16

NA: Not available; ITDA: Integrated Tribal Development Agency; Personal communication: ^; RAAB Repository:@; Population Based Studies:#

*SOURCE: http://data.worldbank.org/indicator/SH.XPD.PCAP?page=3;** SOURCE: http://data.un.org/CountryProfile.aspx?crName=MYANMAR

**Table 2:** Cataract Surgical Coverage (by eyes), stratified by gender

Country	Location	Year	Eyes	Eyes	Eyes	Eyes	Eyes	Eyes	Eyes	Eyes	Eyes	GDP at time of survey*	Per capita health expenditure**
			**Less than 3/60**			**Less than 6/60**			**Less than 6/18**				
			**Males**	**Females**	**Total**	**Males**	**Female**	**Total**	**Males**	**Females**	**Total**		
India^	15 districts In 16 states	2007	NA	NA	62.9	NA	NA	47.7	NA	NA	NA	$1.23 trillion	$43
India	Nandurbar^9^	2009	NA	NA	NA	NA	NA	9.4	NA	NA	NA	$1.36 trillion	$48
India	Kolar^10^	2011	72.1	67.8	69.6	60	57.3	58.4	50	48.6	49.2	$1.83 trillion	$66
India	Sindhudrug^11^	2010	NA	NA	NA	NA	NA	NA	NA	NA	NA	$1.17 trillion	$59
India	Slvaganga^7^	1999	NA	NA	NA	NA	NA	NA	NA	NA	NA	$466.86 billion	$18
India^	ITDA-Khammam & Warngal	2009	71.2	68.5	69.6	61.4	61.4	61.4	45.5	50.9	48.6	$1.36 trillion	$48
India^	ITDA-East Godavari & West Godavari	2009	68.8	65.1	66.5	60.1	57.3	58.4	41.8	42.4	42	$1.36 trillion	$48
India^	ITDA-Srisail-am	2009	75.2	72.3	73.6	68	65.2	66.5	49.2	45.4	47.1	$1.36 trillion	$48
India@	Tribal region In Su rat Gujarat	2011	89	82.2	84.9	77.7	69.1	72.5	48.1	42.1	44.5	$1.83 trillion	$66
Bangladesh	Satkhira^12^	2005	34.6	34.9	34.8	30.9	30.4	30.6	17.4	18.7	18.1	$69.44 billion	$12
Bangladesh	8 districts^13^	2010	61.5	49.7	55.1	38.2	30.9	33.9	20.1	21.3	22.9	$115.27 billion	$23
Bhutan [Urban]	Whole country^5^	2005	65.1	69.8	67.5	61.2	57.1	59	40.6	38.6	39.5	$818.86 billion	$66
Bhutan [Rural]	Whole country	2005	59.7	42.6	50.9	44.3	31.4	37.6	27.9	19.5	23.5	$818.86 billion	$66
Bhutan [Both]	Whole country	2005	61.5	51.3	56.3	49	38.9	43.7	31.3	24.8	27.9	$818.86 billion	$66
Srilanka [40 yrs above and bleow]#	Kandy^14^	2006	67.2	63.6	65.2	60	60.5	60.3	35.1	33.1	34	$28.27 billion	$58
Nepal@	country	2008-2010	68.9	65.7	67.1	59.5	56.6	57.9	40	38.8	39.4	$12.54 billion	$29
Pakistan#	National^8^	2003-2005	64.5	58.4	61.4	54.5	50.0	52.2	42.8	36.6	40.7	$83.24 billion	$16

NA: Not available; ITDA: Integrated Tribal Development Agency; Personal communication: ^; RAAB Repository:@; Population Based Studies:#

*SOURCE: http://data.worldbank.org/indicator/SH.XPD.PCAP?page=3; ** SOURCE: http://data.un.org/CountryProfile.aspx?crName=MYANMAR
